# A Topical Collection on ICT for Health Science Research – EFMI Special Topic Conference

**DOI:** 10.1007/s10916-021-01739-2

**Published:** 2021-05-17

**Authors:** Thomas M. Deserno, Martin Dugas, Matthias Löbe, Jürgen Stausberg

**Affiliations:** 1 Peter L. Reichertz Institute for Medical Informatics of TU Braunschweig and Hannover Medical School, Braunschweig, Germany; 2 Institute of Medical Informatics, University Hospital Heidelberg, Heidelberg, Germany; 3 Institute for Medical Informatics, Statistics and Epidemiology (IMISE), University of Leipzig, Leipzig, Germany; 4 Institute for Medical Informatics, Biometry and Epidemiology (IMIBE), Faculty of Medicine, University Duisburg-Essen, Essen, Germany

Medical Informatics (or health informatics) is considered the science of applying the methods of computer science to health-care. In particular, medical informatics provides methodologies for systematic organization, representation, and analytics of data that is collected in health and well-being. About 50 years ago, the goal has been worded by Peter L. Reichertz: “the right information at the right place at the right time”. In this regard, the European Federation for Medical Informatics (EFMI), which is composed of national member societies, such as the German Association for Medical Informatics, Biometry and Epidemiology (GMDS), organizes annually a special topic conference (STC) that specialize in current trends in medical informatics.

The 2019 edition of EFMI STC was focused on information and communication technology (ICT) for Health Science Research. A major challenge in this field is the syntactical and semantical integration of ICT systems, since much of the data for health science research is coming from healthcare. Nevertheless, research often requires data of higher resolution, precision, and quality than is typically available in healthcare ICT systems. Thus, healthcare data are extracted, transformed, and loaded into research data warehouses, which leads to duplication of data and might challenge data integrity from specific individuals across research and healthcare systems, possibly hindering personalized medicine and translational research. ICT systems for health science research are used in application domains such as clinical trials, development of drugs and medical devices, as well as translational medicine, aiming at better prevention, diagnostics, and interventions in health and care. In addition, ethical, legal, and social aspects of health data are considered.

EFMI STC 2019 was held at the Peter L. Reichertz Institute for Medical Informatics of TU Braunschweig and Hannover Medical School in Hanover, Germany. It was jointly organized with the celebration of the 50th anniversary of the appointment of Peter L. Reichertz to the Hannover Medical School, which founded medical informatics as a research field in Germany in 1969. From the total of 87 paper submissions, the scientific program committee (SPC) – which was strictly different from the local organizing committee (LOC) – selected 48 oral and 22 poster presentations, which have been published in the conference proceedings [1]. However, the authors could decide to publish only an abstract within the proceedings and submit the extended paper to this special topic at JOMS.

 Seven of these submissions finally have passed the strict peer-review process, which, due to the Conora pandemic, lasts almost two years. These papers focus on research and development of information systems supporting biomedical, translational, and clinical research, as well as interoperability across such systems for the purpose of data integration, improving findability, and supporting analytics of cross-system data. They all have been submitted before the pandemic, but have been published during the pandemic. Therefore, we partly reflect them in the light of COVID-19, too.

## Pioneering medical informatics – is history still relevant?

“Professor Peter L. Reichertz is one of the most significant pioneers in the field of medical informatics worldwide.” This quote is taken from Haux [2], the first sentence of a paper, where the author tries to repute the work of Reichertz, who passed away already in 1987. Over the years, there is a clear shift from medical topics – as Reichertz was physician in internal medicine – towards medical informatics and computer science (except medical informatics) in his later work. The topics focus on (i) applications for diagnosis and decision support, (ii) hospital information systems, and (iii) information systems for outpatient care. The literature analysis is concluded by stating that Reichertz – unlike many others – did not only use ICT as if they were like microscopes or ultrasonic devices, but that he was “… visionary enough to very early see the revolutionary potential of informatics for all aspects of biomedicine and healthcare.” [2]. And what has been missing in the early phase of the pandemic? Information systems such as apps that track inter-human contacts [3], or semantically interoperable data sets, such as the German Corona Consensus Data Set (GECCO) [4], both according to Reichertz’s visions.

## eHealth as game-changer of health services research

Health services research has benefitted from the increasing availability of electronically available administrative data for a period of 50 years [5, 6]. Administrative data can be used to describe patterns of healthcare without efforts for data acquisition. Furthermore, a standardization within a healthcare system is guaranteed through nationally defined regulations. Van Laere et al. go a step further [7]. Their research started with electronically available data about prescriptions. Such data has been available for years in Germany, for example, based on a collection of paper-based prescriptions from pharmacies or downstream data centers. As many other countries, Belgium strives for a complete dematerialization of the prescription process (ePrescription). On the one hand, more information about healthcare becomes accessible, making analyses possible on a broader as well as a more detailed level. On the other hand, metadata about electronic services enable health services research to evaluate electronic processes. Van Laere et al. used metadata about the behavior of pharmacies to identify problems in the prescription process as well as errors in the underlying ICT infrastructure. Thereby, health services research got a double role in analyzing the patterns of ePrescriptions in Belgium, not only investigating the daily routine of electronic services in healthcare, but also evaluating the digitization itself. Interestingly, the latter was responsible for more than a third of the reasons for treating ePrescriptions as paper-based by Belgian community pharmacists. However, the new opportunity for health services research creates special challenges. For example, the case of interoperability must be extended to technical metadata [8] about electronic services in healthcare. Furthermore, the community behind health services research must be empowered to understand digitization issues, not only to understand healthcare. In the light of the pandemic, pharmacists may become more active in vaccine administration, as – among others – European countries have already adopted their legal role [9]. Therefore, ICT for health science research remains a challenge for both perspectives, for enablers providing the technology and for researchers analyzing patterns that reflect healthcare as well as technological aspects.

## Patients as stakeholders in health science research

Traditionally, patients had the role of an observational unit in empirical health science research. This paradigm has changed. On the one hand, the patients’ perspective about effects of interventions got more attention in terms of patient-related outcome measures (PROMs) [10]. Measuring the effect of interventions on quality of life is one example. On the other hand, patients get involved in the planning, the design, and the management of research projects. With this regard, Rauter and colleagues [11] analyzed the perspective of German patient organizations (PO) towards their involvement as stakeholders in research projects. They identified four different roles of POs: mediator, cooperator, financer, and independent. Moreover, Rauter and colleagues pointed out that “involvement” addresses different levels of detail, ranging from the passive consent to the role of a sponsor. However, patients as stakeholders do not necessarily need POs as intermediate. Patients themselves create research platforms and contribute actively and independently from academic institutions to health science research [12]. Consequently, the US-American Agency for Healthcare Research and Quality (AHRQ) called the academic community to move to patient-centered care and patient-centered research [13]. The AHRQ divided the involvement of patients in four categories: increasing focus on the patient, engaging patients as partners, digital health with patient reported data and automatic vital signs recordings, and direct-to-patient activities, e.g., by self-recruitment of patients. These trends create new challenges for health sciences. For instance, the management of ten or twenty study sites in a clinical trial may turn towards thousands of self-recruited patients who act as observational unit as well as study site. Monitoring and verification of PRO with query alerts fail if data recording is up to the patients in their daily life [14]. The work of Rauter and colleagues is an important contribution to understand the challenges of patient-centered research, even more, since PROM has been established as method and means after Covid-19 disease [15, 16].

## Automatic recording of patient’s health parameters

However, measurements on patients not only include vital signs. The World Health Organization (WHO) defines environmental, behavioral, physiological, and psychological parameters to assess the quality of life [17, 18]. A crucial point is the automatic assessment of psychological parameters, which by nature can be captured only indirectly. Ganapathy et al. measure the electrodermal activity to estimate the emotional state of the subject [19]. They propose a multiscale deep convolutional neural network to score valence and arousal each into two groups of low and high level, according to Russell’s two-dimensional emotion space [20]. The number of layers and the signal length are the determinants for the classifier performance. The approach provides end-to-end learning and classification of emotional states without additional signal processing. Such method can further be useful to assess the emotional states and their tendencies, in particular when applied to children suffering from pandemic threat, quarantine, and social distancing [21].

## Focus on interoperability in clinical information systems

Standards play an important role in research, especially in the area of interoperability. But good standards do not only impress by a good specification and a high acceptance in the scientific community, they also prove their importance and usability by practical implementation. One recent approach for achieving interoperability are Health Level Seven (HL7) Fast Healthcare Interoperability Resources (FHIR). The main goal of FHIR is to build a versatile platform that can implement common scenarios, but without getting lost in epic details. The extensible open-source imaging informatics software platform (XNAT), on the other hand, is a web-based software for archiving, managing, and sharing medical images and associated data in a research context. It is used for central research data repositories as well as for multicenter studies. XNAT can handle Digital Imaging and Communications in Medicine (DICOM) data and generally utilize the eXtensible Markup Language (XML). Khvastova et. al. address the question of enabling XNAT to work with FHIR data using the *patient* artifact as an example [22]. Patients are a central construct in many information systems of subject-oriented research, largely containing demographic and project administrative data. The authors developed an XNAT plugin that maps data elements from FHIR to their corresponding parts in the XNAT schema. By doing so, they not only improve accessibility of the data and interoperability between software instances. They also improve semantics because of the characteristic of FHIR to heavily rely on external terminologies like the Systematized Nomenclature of Medicine – Clinical Terms (SNOMED CT). These are important aspects of FAIR data [23, 24], too. On the other hand, the approach would not have been possible if the developers of XNAT had not chosen a modern, open-source architecture that offers both Representational State Transfer (REST) interfaces and the ability to extend the internal data format with custom data types. This shows the necessity in the design of information systems not to see them as isolated solutions that can serve exactly only the originally intended purpose. It is easy to imagine the advantages in this case of an automated and updatable transfer of patient master data in terms of workload reduction and data quality for larger studies, as it became necessary for other scenarios in the wake of the COVID-19 epidemic.

## New approaches in clinical research informatics

Varghese et al. report about a study portal to visualize the geographic distribution of study research networks [25]. This system helps to find clinical studies for a specific disease in the vicinity of a certain location. The portal applies public data and semantic annotation. It is another medical informatics approach to support clinical studies with new digital health systems. Of note, patient recruitment is a well-known challenge for clinical research: on a global perspective, only about one third of clinical trials can be completed on time. The COVID-19 pandemic clearly demonstrated that speed matters in medical research. Thus, routine care and research need to be much more connected to establish a learning health system. Open metadata and data integration (data FAIRification [24]) are key success factors to leverage information systems to speed-up and improve clinical research.

## Common data models in practice

The question of interoperability holds also for database schemas. A natural approach is to design a database schema oriented to the needs of the specific use case. Schema designs often refine over time to reflect new requirements and thus become more complex. Common data models (CDMs) are schemas that claim to cover a broader range of use cases. They are based on a model that has been consented in a circle of experts and developed over a longer period of time. Although new functionalities are added to CDMs, their core is often stable. This leads to the development of an ecosystem of different tools from the user community. However, the major advantage of using a CDM is the uniformity of data in distributed repositories. This allows for distributed, reproducible analyses across multiple sites from different regions or domains with large numbers of subject and thus tends to have greater statistical power. Furthermore, the clearly defined semantics allow results from different studies to be compared. If existing data is to be transferred to a CDM, complex schema matching is necessary. This process is called data standardization. Haberson et al. [26] examine to what extent the Observational Medical Outcomes Partnership (OMOP) CDM is suitable to represent the data of the Austrian Health Claims Database GAP-DRG, which contains entries from 95% of the population. For their purpose, they select a manageable subset of the data. The actual transformation is preceded by a vocabulary mapping, since OMOP is based on standard medical terminologies like the International Classification of Diseases (ICD) or SNOMED CT and the locally used catalogs have to be mapped to the preferred universal concepts, which can be semantically challenging. As a result, certain variables cannot be mapped if, for example, the algorithmic basis differs too much. Although the proof of feasibility has not yet been demonstrated beyond doubt and requires further research, a plea for the use of international terminologies that would make such mapping steps obsolete can be gleaned from the issues described.

## Lessons learned

In conclusions, ICT for health science research is an ongoing field, and several new aspects have been added due to the current pandemic. Still, there is a “revolutionary potential of informatics for all aspects of biomedicine and healthcare” [2]. In particular:

- Data matters: FAIR reusable and interoperable data is required to improve health and well-being. This includes further standardization to achieve semantical interoperability between the healthcare providers, not only on a national level, but also internationally, as we are facing today global health crises. In this context, the concept of a “broad consent” [27] becomes further important.
- Security matters: Exchanging health data requires reliable and secure ICT infrastructure, not only for clinical trials but also for contact tracing and tracking. Good medical informatics practice can seed trust such that individuals participate and donate their health data to the public.
- Time matters: After one year of pandemic, still relevant data is transported paper-based. This holds for Germany (e.g., the invitations for vaccination are snail-mailed) as well as for other countries. This causes delays and bias to statistics, false interpretation of data, and wrong actions. Improved ICT can resolve this problem, too.
